# Aqueous humour cytokine profiles after Descemet’s membrane endothelial keratoplasty

**DOI:** 10.1038/s41598-021-96566-3

**Published:** 2021-08-23

**Authors:** Takahiko Hayashi, Hidenori Takahashi, Satoru Inoda, Toshiki Shimizu, Akira Kobayashi, Hidetoshi Kawashima, Takefumi Yamaguchi, Satoru Yamagami

**Affiliations:** 1 Division of Ophthalmology, Department of Visual Sciences, Nihon University School of Medicine, Ohyaguchikami-machi 30-1, Itabashi-ku, Tokyo, 173-8610 Japan; 2 Department of Ophthalmology, Yokohama Minami Kyosai Hospital, Kanagawa, Japan; 3 Department of Ophthalmology, Yokohama City University School of Medicine, Kanagawa, Japan; 4 Department of Ophthalmology, Jichi Medical University, Tochigi, Japan; 5 Department of Ophthalmology, Graduate School of Medical Science, Kanazawa University, Ishikawa, Japan; 6 Department of Ophthalmology, Tokyo Dental College, Ichikawa General Hospital, Chiba, Japan

**Keywords:** Immunology, Medical research, Optics and photonics

## Abstract

The aim of this study was to compare aqueous humour (AqH) cytokine profiles before and after Descemet’s membrane endothelial keratoplasty (DMEK) in eyes with bullous keratopathy (BK) and age-matched normal eyes. A total of 49 participants (mean age 75.0 ± 13.5 years) were divided into three groups: (1) BK patients scheduled for DMEK (BK group); (2) patients after DMEK (DMEK group; average postoperative time 1,018 ± 460 days); and (3) control participants without corneal endothelial disease scheduled for cataract surgery (control group). AqH (0.2 mL) was collected from all participants, and the levels of various AqH cytokines were simultaneously measured using a multiplex bead immunoassay. The DMEK group exhibited significantly lower concentrations of several pro-inflammatory cytokines, such as IL-1β, IL-5, IL-6, IL-10, and IL-8, and granulocyte colony stimulating factor than the BK group. In addition, the levels of IL-1β and IL-5 were significantly lower in the DMEK group than in the control group. The AqH levels of several pro-inflammatory cytokines were significantly reduced after DMEK, indicating that regeneration of the endothelial pump owing to the transplantation of a healthy endothelium might have a positive effect on anterior chamber inflammation.

## Introduction

Descemet’s membrane endothelial keratoplasty (DMEK), which was introduced by Dr. Gerrit Melles, has become the gold standard for the surgical management of corneal endothelial disease^1–3^. DMEK is a minimally invasive surgical procedure, where only the Descemet’s membrane together with the endothelium, but without any stromal components, is exchanged^4–10^. Compared with other keratoplasty techniques, DMEK displays several advantages, such as excellent and quick visual recovery and a very low rate of allograft rejection^11–13^.

Despite these merits, there is a significant post-operative decrease in corneal endothelial cell density (ECD) over the years. As insufficient ECD inevitably leads to graft failure, the characterisation of mechanisms underlying post-operative ECD loss is of outmost importance^14–16^. Recently, we have demonstrated a strong correlation between aqueous humour (AqH) inflammation and ECD loss following corneal transplantation^17–23^. In addition, Lužnik et al. have reported increased levels of the pro-inflammatory cytokine, interleukin (IL)-8, in the AqH of failed DMEK cases, suggesting that innate immune activation may exert an adverse influence on ECD^24^. Furthermore, we have recently reported the regression of corneal neovascularisation (which is often associated with severe corneal inflammation) following DMEK^25^, indicating that healthy corneal endothelial cells (CEnCs) may play a pivotal role in modulating inflammatory conditions in the cornea.

Thus, although AqH inflammation seems to be an important factor that facilitates a decrease in ECD following DMEK, post-operative AqH cytokine profiles associated with this process have not yet been evaluated. We hypothesised that DMEK surgery ameliorates AqH inflammation by providing healthy CEnCs and, thereby, lowering the levels of inflammatory cytokines. To test this hypothesis, we characterised the cytokine profile in the AqH of eyes before and after DMEK. To the best of our knowledge, this is the first study to analyse AqH in eyes that are in a stable state following DMEK.

## Results

### Characteristics of the participants

The characteristics of the study patients are summarised in Table 1. The mean age of the patients was 75.0 years (range, 54–89 years), and 14 of the 49 patients (28.6%) were males. Mean axial length (AXL) was 23.6 ± 1.4 mm, and mean intraocular pressure (IOP) was 13.6 ± 3.2 mmHg. The reasons for endothelial dysfunction were pseudophakic bullous keratopathy (12 eyes in the BK group, 12 eyes in the DMEK group), Fuchs endothelial corneal dystrophy (two eyes in the BK group; one eye in the DMEK group), pseudoexfoliation syndrome (one eye in the BK group, one eye in the DMEK group), and chemical burn (one eye in the BK group).

**Table 1:** Participant characteristics.

	BK	DMEK	Control	*P* value (BK-CT)	*P* value (BK-DMEK)	*P* value (DMEK-CT)
N, eyes	16	14	19			
Age (SD), years	76.75 (8.30)	75.43 (7.95)	72.89 (8.45)	0.43	0.99	0.99
Sex, male (%)	4 (25)	1 (7.2)	9 (47.4)	0.17	0.19	**0.013**
BCVA (SD), (LogMAR)	0.98 (0.66)	0.06 (0.14)	0.14 (0.42)	**< 0.001**	**< 0.001**	0.95
AXL (SD), mm	23.05 (1.40)	23.48 (1.45)	24.29 (1.27)	**0.013**	0.99	0.13
IOP (SD), mmHg	13.13 (3.48)	13.36 (3.43)	14.24 (3.43)	0.10	1.00	1.00
CCT (SD), µm	659 (64.9)	526 (56.9)	533 (21.4)	**< 0.001**	**< 0.001**	0.99

BK, bullous keratopathy; CT, control; DMEK, Descemet’s membrane endothelial keratoplasty; BCVA, best-corrected visual acuity; IOP, intraocular pressure; AXL, axial length; CCT, central corneal thickness.

In the DMEK group, the time from DMEK surgery until AqH collection was 1,018 ± 460 days. In all patients, the indication for DMEK was pseudophakic BK. All patients in the DMEK group had been using low-dose steroid eye drops (fluorometholone 0.1%; Santen, twice a day). The best-corrected visual acuity (BCVA) was 0.06 ± 0.04 logarithm of the minimum angle of resolution (logMAR). In the DMEK group, corneal ECD on the day of AqH collection was 979 ± 357 cells/mm^2^. One eye showed allograft rejection on the day of AqH collection and was excluded from further statistical analyses. In the BK group, all patients were on the waiting list for DMEK surgery. Some patients had been using topical steroids (n = 10) or topical non-steroidal anti-inflammatory drugs (NSAIDs) (n = 1). All eyes in the control group were scheduled for cataract surgery, and none of the patients had been using anti-inflammatory drugs, such as steroids or NSAIDs. In the DMEK group, no clinical feature of anterior chamber inflammation (keratic precipitates [KPs], synechiae, or flare) was detected except for one eye with graft rejection. On the contrary, most of BK patients showed some KPs although the clear picture was not preserved owing to oedematous and hazy cornea. There was no inflamed eye in the control group.

All data in our cohort are shown as a dataset (Supplementary Data set).

### Aqueous protein levels

**Figure 1:**
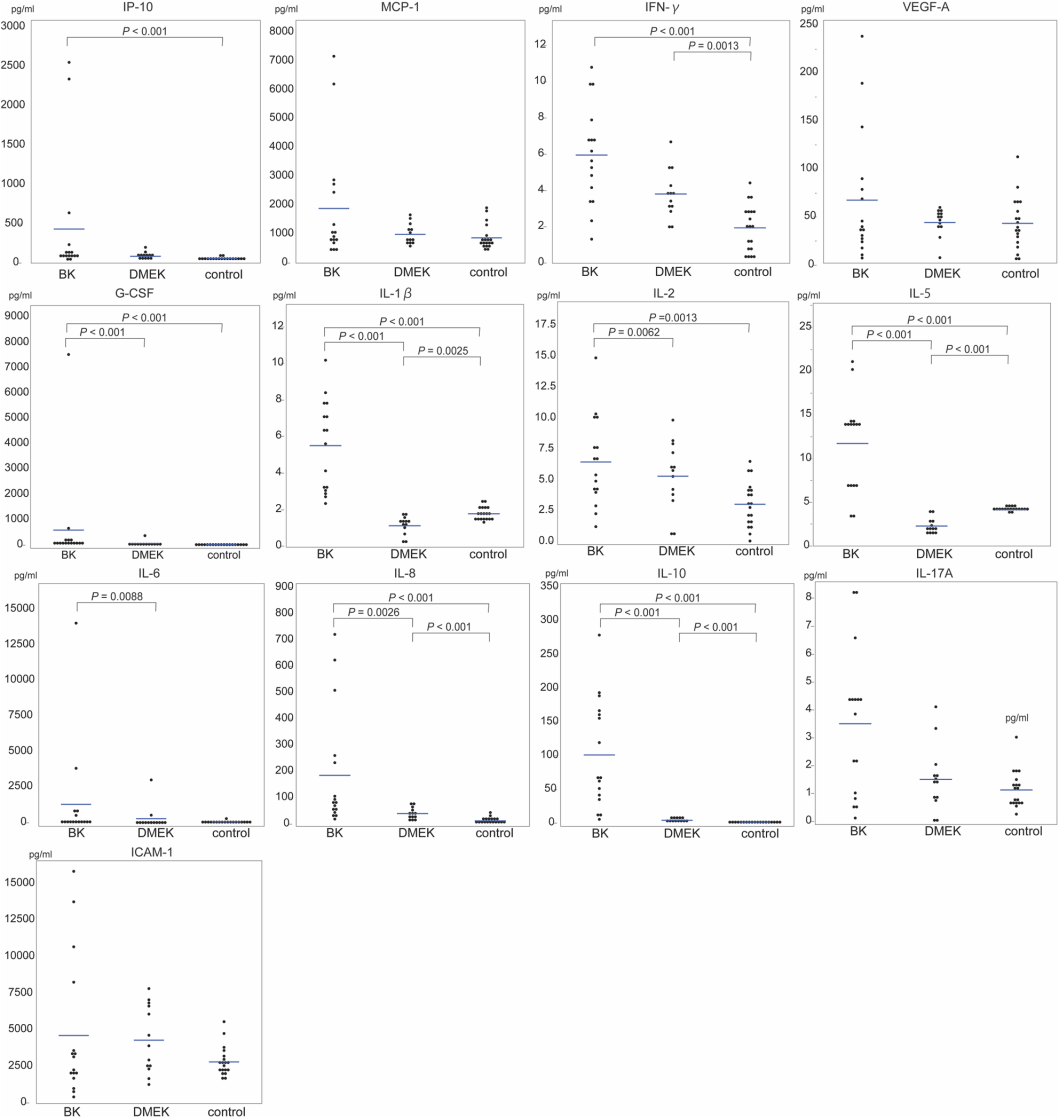
Inflammatory cytokine concentrations. The cytokine concentrations of interferon gamma-induced protein [IP]-10, monocyte chemotactic protein [MCP]-1, interferon [IFN]-γ, vascular endothelial growth factor-a [VEGF-A], granulocyte colony stimulating factor [G-CSF], interleukin [IL]-1β, IL-2, IL-5, IL-6, IL-8, IL-10, IL-17A, and the intercellular adhesion molecule [ICAM-1] in AqH were measured using a multiplex bead immunoassay, and compared among BK, DMEK, and control groups. Cytokine concentrations were logarithmically transformed and analysed using Tukey’s test. Note the different scaling of the y-axis.

AqH cytokine concentrations for all analysed groups are shown in Supplementary Table S1 and in Fig. 1. The concentrations of interferon gamma-induced protein (IP-10), interferon (IFN)-γ, granulocyte colony stimulating factor (G-CSF), interleukin (IL)-1β, IL-2, IL-5, IL-8, and IL-10 in the BK group were significantly higher compared to those in the control group (all *p* values < 0.01). No significant differences were detected in the concentrations of monocyte chemotactic protein (MCP-1), soluble intercellular adhesion molecule (ICAM)-1, IL-6, IL-17A, and vascular endothelial growth factor (VEGF)-A between the BK and control groups (all *p* values > 0.05).

The AqH concentrations of G-CSF (*p* < 0.001), IL-1β (*p* < 0.001), IL-2 (*p* = 0.0062), IL-5 (*p* < 0.001), IL-6 (*p* = 0.0088), IL-8 (*p* = 0.0026), and IL-10 (*p* < 0.001) were significantly lower in the DMEK group than in the BK group. The AqH concentrations of IP-10, MCP-1, IFN-γ, VEGF-A, IL-17A, and ICAM-1 were also lower in the DMEK group than in the BK group, although no statistically significant differences were observed for these factors.

The AqH concentrations of IFN-γ (*p* = 0.0013), IL-8 (*p* < 0.001), and IL-10 (*p* < 0.001) in the DMEK group were significantly higher than those in the control group, whereas the AqH concentrations of IL-1β (*p* = 0.0025) and IL-5 (*p* < 0.001) were significantly lower in the DMEK group. For IP-10, MCP-1, VEGF-A, G-CSF, IL-2, IL-6, IL-17A, and ICAM-1, no statistically significant difference between the DMEK and control group could be detected (all *p* values > 0.05).

In addition, AqH cytokine levels in one eye that was newly diagnosed with graft rejection after DMEK were measured. This eye showed high cytokine levels of IP-10 (6247.25 pg/mL; compared to a mean of 18.77 pg/mL in the control group), G-CSF (1568.14 pg/mL, compared to a mean of 10.51 pg/mL in the control group), MCP-1 (3158.59 pg/mL, compared to a mean of 866.9 pg/mL in the control group), IFN-γ (13.37 pg/mL, compared to a mean of 1.94 pg/mL in the control group), and IL-6 (29837.1 pg/mL, compared to a mean of 54.8 pg/mL in the control group).

## Discussion

In this study, we evaluated the cytokine profiles of IL-2, IFN-γ, ICAM-1, IL-5, IL-6, IL-8, IL-10, IL-1β, MCP-1, IL-17A, IP-10, G-CSF, and VEGF-A in the AqH of BK patients before and after DMEK and in a control group without corneal disease.

Our results demonstrate that the AqH concentrations of several inflammatory cytokines, such as IP-10, IFN-γ, G-CSF, IL-1β, IL-2, IL-5, IL-8, and IL-10, in the BK group were significantly higher than in the control group (Fig. 1), which is consistent with previous studies^17–23^.

A major strength of this study is the post-operative evaluation of AqH cytokine profiles in eyes that underwent DMEK. Although the existence of a correlation between immune responses and graft failure following DMEK has been reported^24^, no comprehensive evaluation of AqH cytokine profiles following DMEK has been performed, especially during the stable and quiescent post-operative period. The average duration of the post-operative period in the DMEK group was 1,018 ± 460 days, which is a relatively long-term follow-up after DMEK. Our results showed that the concentrations of some AqH cytokines (IFN-γ, IL-8, and IL-10) in the DMEK group were significantly higher than in the control group (Fig. 1), whereas the concentrations of other cytokines (IL-1β and IL-5) were significantly lower in the DMEK group. The analysis of eight other factors (IP-10, MCP-1, VEGF-A, G-CSF, IL-2, IL-6, IL-17A, and ICAM-1) did not reveal any statistical difference between the DMEK and control groups, indicating that DMEK surgery is associated with a complex change in the anterior chamber environment.

**Figure 2:**
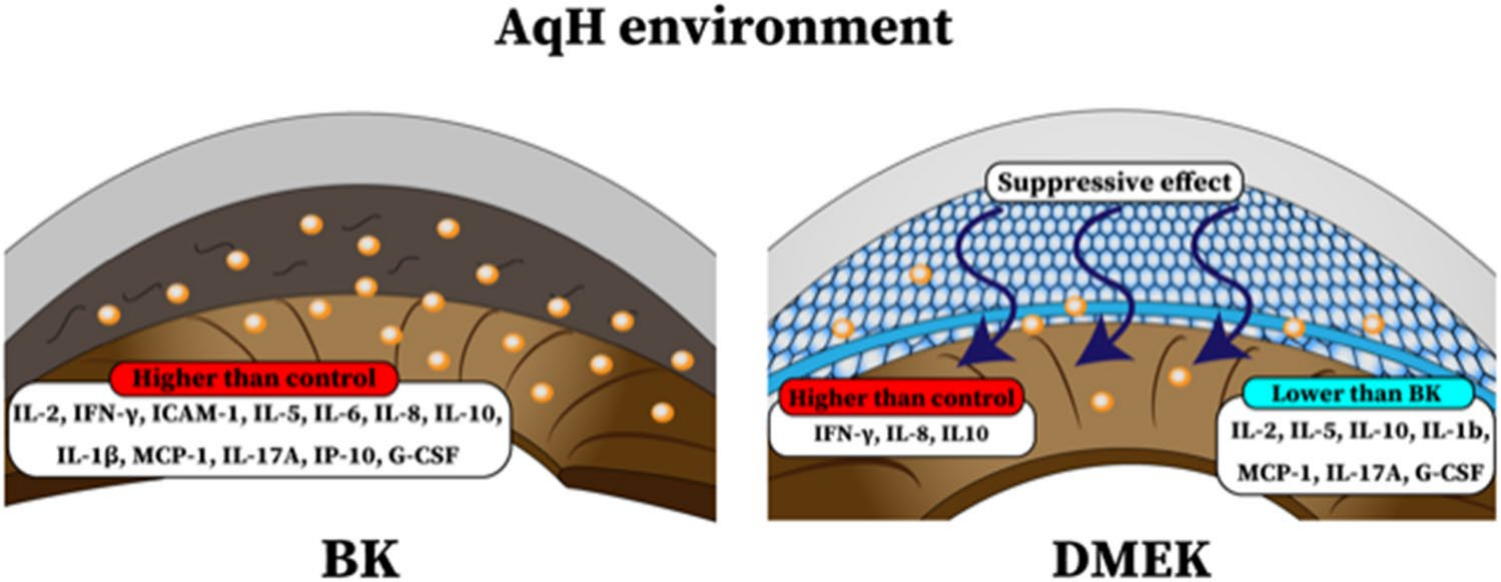
Cytokine concentrations in BK and DMEK eyes. (**a**) Eyes with bullous keratopathy (BK) show increased AqH concentrations of various inflammatory cytokines when compared with healthy eyes, possibly due to a lack of protective/suppressive effects of corneal endothelial cells (CEnCs). (**b**) After DMEK, cytokine levels are suppressed in eyes with BK, arguably by transplanted CEnCs, although levels of some cytokines after DMEK are still higher when compared with those in healthy eyes. This image was illustrated by our co-author (Toshiki Shimizu).

Importantly, the concentrations of all the analysed cytokines were remarkably lower in the DMEK group than in the BK group, most of which were statistically significant (Fig. 1). This finding clearly demonstrates that the transplanted healthy endothelium has a positive effect on anterior chamber inflammation in eyes with BK. Previous studies have indicated that CEnCs play an important role in maintaining the immune-privileged status of the cornea (Fig. 2)^26,27^. Thus, the higher concentrations of inflammatory cytokines observed in eyes with BK may be attributable to the absence of healthy and protective CEnCs. After transplantation, transplanted healthy CEnCs might inhibit these inflammatory cytokines and restore the silent anterior chamber environment. Therefore, any surgical method providing healthy corneal endothelium, such as PKP and Descemet stripping automated endothelial keratoplasty (DSAEK), might show the similar effects on the anterior chamber inflammation. CEnCs may play an important role in anterior chamber inflammation and immunity, for example, via the programmed death-1 (PD-1)/B7-H1 co-stimulatory pathways^26^, or vasoactive intestinal peptides^28^.

Interestingly, we obtained cytokine profiles from a patient presenting with allograft rejection following DMEK. We detected relatively high levels of several inflammatory cytokines, including IP-10, G-CSF, MCP-1, IFN-γ, and IL-6. IP-10 is induced by IFN-γ, which is related to the Th1 pathway^29^, whereas G-CSF, MCP-1, and IL-6 are associated with macrophages^30^. Notably, the up-regulation of IL-6 levels has already been reported in cases presenting with allograft rejection following penetrating keratoplasty (PKP)^31^. Despite the differences between PKP and DMEK, our results are consistent with those of this study.

Some of our study subjects had used topical steroids prior to the collection of AqH. However, we did not detect any significant difference between the concentration of cytokines in the AqH of eyes treated with topical steroids and those that were not treated^19,20^. In fact, concentrations of some cytokines were higher in eyes treated with topical steroids than in those that were not. Although topical steroids play an essential role in preventing allograft rejection^32^, our results also show that high AqH concentrations of at least some inflammatory cytokines remain in the eyes after uneventful DMEK, regardless of the use of low-dose steroid eye drops. Several studies have described the positive effects of using topical steroids and NSAIDs in endothelial keratoplasty^33,34^; thus, proper treatments that reduce inflammatory responses following DMEK, such as steroids, NSAIDs, or ‘anti-cytokine’ eye drops, should certainly be considered.

Our study was beset with some limitations, such as the relatively small number of patients and the lack of evaluation of flare by laser flare-cell photometry, or of a cytokine profile time course following DMEK. Moreover, future prospective studies are necessary to compare changes in cytokine levels at earlier phases (1, 3, 6, and 12 months) after different types of keratoplasty, such as PKP, DSAEK, and DMEK.

Taken together, our current study clearly demonstrates that after DMEK, significant changes in AqH cytokine profiles occur. These changes may be attributable to the immunomodulatory effect of transplanted CEnCs. As DMEK may be useful to reduce anterior chamber inflammation in patients with BK, early intervention should be considered in treating inflammation in these eyes. However, a certain level of inflammation persists even after uneventful DMEK (compared to healthy eyes without corneal transplants), which might warrant the development of specific anti-inflammatory drugs that endow patients with long-term graft survival.

## Methods

### Study design and approval

The current cross-sectional study was approved by the Institutional Review Board of Jichi Medical University and Yokohama Minami Kyosai Hospital (YKH29_11_2, JICHI18-019), and adhered to the tenets of the Declaration of Helsinki. The study was registered as a clinical trial (UMIN000020718). The experimental procedures followed all institutional guidelines, and written informed consent was obtained from all patients before each procedure.

### Collection of AqH

Between September 1 and September 30, 2020, we collected AqH (0.2 mL per participant) at Yokohama Minami Kyosai Hospital from all study participants. Briefly, after the sterilisation of eyes with an antiseptic agent (povidone-iodine), approximately 0.2 mL of AqH was collected under a surgical microscope using a 30-gauge needle. The samples were immediately stored at − 80 °C until the proteins were analysed.

The participants were divided into three groups: (1) eyes with BK scheduled for DMEK (BK group), (2) eyes that had previously received DMEK (DMEK group), and (3) control eyes without any signs of corneal disease scheduled for cataract surgery (control group). Patients with additional ocular diseases, including glaucoma, retinal diseases, or uveitis, were excluded from the study^35^. Post-operatively, 1.5% levofloxacin (Cravit; Santen) was administered four times daily for 1 week.

### Measurement of protein concentrations

We analysed AqH protein concentrations of IL-2, IFN-γ, ICAM-1, IL-5, IL-6, IL-8, IL-10, IL-1β, MCP-1, IL-17A, IP-10, G-CSF, and VEGF-A using a multiplex cytokine assay (Filgen, Aichi, Japan) according to the manufacturer’s instructions^36^. All samples were tested simultaneously. The samples were measured twice at each time point, and the average score was calculated.

### Ophthalmic examinations

In all participants, BCVA, logMAR, central corneal thickness (CCT), ECD, AXL, and IOP were measured prior to the collection of AqH. BCVA was measured as decimal visual acuity and converted to logMAR units for statistical analysis. CCT was measured using corneal tomography (SS1000; Tomey Corporation, Aichi, Japan) and evaluated by a corneal specialist (HT). ECD was evaluated using a specular microscope (FA3509; Konan Medical Hyogo, Japan). AXL was measured via optical biometry (IOL Master 500, Carl Zeiss Meditec, Oberkochen, Germany).

### Statistical analysis

Statistical analyses were performed using the JMP Pro software (version 13.2.0; SAS Institute, Cary, NC, USA). The Kruskal–Wallis test with Dunn’s multiple comparisons test was used to compare age, BCVA, AXL, IOP, and CCT among the three groups. Nominal variables, such as sex, were compared using Pearson’s chi-square test. Cytokine concentrations were analysed using ANOVA followed by Tukey’s test, after logarithmic transformation because these data showed a lognormal distribution^27^. Statistical significance was set at *p* < 0.05.

## Supplementary Information

Supplementary Information 1.

Supplementary Information 2.

## Data Availability

All data generated or analysed during this study are included in this published article and in its Supplementary Information files.
