# Supplementary material for: Aqueous humour cytokine profiles after Descemet’s membrane endothelial keratoplasty
Source: Sci Rep. 2021 Aug 23;11:17064. doi: 10.1038/s41598-021-96566-3 (PMC8382754; doi:10.1038/s41598-021-96566-3)
Supplement: Supplementary file 2 — Supplementary Information 2. [file 41598_2021_96566_MOESM2_ESM.docx]

**Supplemental Table S1**. Inflammatory cytokine concentration

|  | BK  Mean [95% CI]; pg/mL | DMEK,  Mean [95% CI]; pg/mL | Control,  Mean [95% CI]; pg/mL | p-Value  (BK-CT) | p-Value  (BK-DMEK) | p-Value  (DMEK-CT) |
| --- | --- | --- | --- | --- | --- | --- |
| IP-10 | 393 [-32.87 to 818.8] | 46.48 [24.1 to 68.86] | 18.77 [13.75 to 23.78] | **<0.001** | 0.47 | 0.097 |
| MCP-1 | 1884 [798.6 to 2970] | 987.4 [782.6 to 1192] | 866.9 [666 to 1067] | 0.072 | 0.40 | 0.72 |
| IFN-γ | 5.95 [4.49 to 7.42] | 3.81 [3.04 to 4.58] | 1.94 [1.34 to 2.54] | **<0.001** | 0.30 | **0.0013** |
| VEGF-A | 62.44 [26.54 to 98.35] | 39.08 [31.29 to 46.87] | 38.22 [25.53 to 50.92] | 0.66 | 0.99 | 0.76 |
| G-CSF | 585.1 [-403.9 to 1574] | 55.53 [-1.025 to 112] | 10.51 [8.89 to 12.14] | **<0.001** | **<0.001** | 0.34 |
| IL-1β | 5.49 [4.17 to 6.80] | 1.11 [0.83 to 1.39] | 1.76 [1.59 to 1.93] | **<0.001** | **<0.001** | **0.0025** |
| IL-2 | 6.45 [4.55 to 8.35] | 5.30 [3.71 to 6.89] | 3.03 [2.19 to 3.88] | **0.0013** | **0.0062** | 0.98 |
| IL-5 | 11.7 [8.85 to 14.56] | 2.26 [1.76 to 2.76] | 4.12 [4.04 to 4.20] | **<0.001** | **<0.001** | **<0.001** |
| IL-6 | 1,292 [-588.3 to 3,173] | 280.1 [-200.9 to 761.2] | 54.84 [22.23 to 87.46] | 0.064 | **0.0088** | 0.55 |
| IL-8 | 184.8 [63.22 to 306.5] | 39.68 [27.09 to 52.27] | 11.83 [8.31 to 15.37] | **<0.001** | **0.0026** | **<0.001** |
| IL-10 | 100.9 [57.87 to 143.9] | 3.98 [3.19 to 4.77] | 1.30 [0.95 to 1.65] | **<0.001** | **<0.001** | **<0.001** |
| IL-17A | 3.491 [2.09 to 4.893] | 1.50 [0.83 to 2.167] | 1.12 [0.80 to 1.43] | 0.084 | 0.069 | 0.95 |
| ICAM-1 | 4,609 [2,047 to 7,171] | 4,290 [2,977 to 5,602] | 2,802 [2,333 to 3,271] | 0.95 | 0.60 | 0.41 |

BK, bullous keratopathy; DMEK, Descemet’s membrane endothelial keratoplasty; CT, control; IL, interleukin; IFN, interferon; ICAM, intercellular adhesion molecule-1; MCP, monocyte chemotactic and activating factor; VEGF, vascular endothelial growth factor. Cytokine concentrations were logarithmically transformed and analysed by Tukey’s *t*-test.

The AqH concentrations of G-CSF (p<0.001), IL-1β (p<0.001), IL-2 (p=0.0062), IL-5 (p<0.001), IL-6 (p=0.0088), IL-8 (p=0.0026), and IL-10 (p<0.001) in the DMEK group were significantly lower than those in the BK group. The AqH concentrations of IFN-γ (p=0.0013), IL-8 (p<0.001), and IL-10 (p<0.001) in the DMEK group were significantly higher than those in the control group, despite the fact that the concentrations of IL-1β (p=0.0025) and IL-5 (p<0.001) were significantly lower in the DMEK group.
